# High relative risk of all-cause mortality attributed to smoking in China: Guangzhou Biobank Cohort Study

**DOI:** 10.1371/journal.pone.0196610

**Published:** 2018-04-26

**Authors:** Tai Hing Lam, Lin Xu, Chao Qiang Jiang, Wei Sen Zhang, Feng Zhu, Ya Li Jin, G. Neil Thomas, Kar Keung Cheng

**Affiliations:** 1 School of Public Health, the University of Hong Kong, Hong Kong SAR, China; 2 School of Public Health, Sun Yat-sen University, Guangzhou, China; 3 Guangzhou No.12 Hospital, Guangzhou, China; 4 Institute of Applied Health Research, University of Birmingham, Birmingham, United Kingdom; Ruhr-Universitat Bochum, GERMANY

## Abstract

**Background:**

Prediction of disease burden in China arising from smoking based on earlier cohorts in the West and China could not reflect the disease burden at the current stage accurately. No cohort studies in China focused specifically on people born since 1950. We examined the risk of all-cause mortality attributed to smoking in adults in Guangzhou, the city with the most rapidly expanding economy in China.

**Methods and findings:**

This population-based prospective cohort included 21,658 women and 8,284 men aged 50+ years enrolled from 2003–2008 and followed until January 2016. During an average follow-up of 8.8 (standard deviation = 1.8) years, 2,986 (1,586 women, 1,400 men) deaths were recorded. After adjustment for confounders, the hazards ratios (95% confidence interval (CI)) of all-cause mortality in current versus never smokers increased from 1.61 (95% CI 1.45–1.80) in those born in 1920–1939 to 2.02 (95% CI 1.74–2.34), and 4.40 (95% CI 3.14–6.17), in those born in the 1940s and 1950s, respectively (P for trend 0.009).

**Conclusions:**

In smokers born after 1949 in Guangzhou and other areas which have the longest history of smoking, the mortality risk could have reached three fold that of non-smokers, as in the UK, US and Australia. If confirmed, unless China quickly and strictly complies with the WHO Framework Convention on Tobacco Control with massive smoking cessation in the population, this is a more striking warning that China will be facing an even larger disease burden from tobacco use than previous forecasts.

## Introduction

Globally, the tobacco epidemic, which refers to an epidemic of tobacco induced mortality and mortality that started with increasing tobacco consumption during the past decades, continues to expand with increasing risk and disease burden especially in middle and low income countries. Previous cohort studies in the early stages of the tobacco epidemic showed consistent under-estimation of mortality risks for cohorts in later stages [1]. Cohort studies a few decades ago from the US and UK observed a two-fold risk of all-cause mortality in smokers relative to non-smokers (i.e. a relative risk (RR) of two). Whilst the prevalence of smoking in these countries has been declining, their RR of mortality in smokers has increased to three (i.e., RR = 3.0, 95% confidence interval (CI) 2.9–3.1, and 3.0, 95% CI 2.7–3.3, respectively) [2, 3], which has also been shown in a recent cohort study in Australia (RR = 3.0, 95% CI 2.7–3.3) [4].

China is considered to be at an earlier stage of the tobacco epidemic [5, 6]. However, emerging evidence has indicated that China may pass through the early stage of epidemic more rapidly, than previous forecasts, with the RR of smoking-induced mortality approaching that in the West. Chen *et al*. comparing the RR in two China nationwide cohorts revealed that the 1991 cohort showed a lower RR but the other cohort, the China Kadoorie Biobank (CKB), with participants recruited in 2004–8 showed a greater RR of 1.65 (95% confidence interval (CI) 1.59–1.73) in urban men and 1.51 (95% CI 1.40–1.63) in women [7]. Such estimations of relative risk that are approaching two as in the West [8], were close to that reported earlier in a small cohort in 1976 in Xi’an, China [9]. The CKB was large, but did not include the largest and most economically developed cities such as Beijing, Shanghai and Guangzhou. Cohorts set up more than20 years ago from Beijing and Shanghai showed an elevated mortality risk of 20–40% from smoking, which may represent underestimates for the more recent cohorts in China [10, 11].

The People’s Republic of China (PRC) was inaugurated in October 1949. Since the1980s, the economic reform has transformed China from one of the world’s poorest country through the world’s most rapid economic development to the second largest global economy. This has been mirrored by a rapidly increasing prevalence of smoking and cigarette consumption mainly in men. As described above, cohort studies on people mostly born before 1950 may show under-estimates of smoking-induced mortality risk and could not assess the rapidly increasing risk because smokers born since 1950 had started smoking cigarettes younger and smoked more [7]. Guangzhou’s economic development, urbanization and westernisation have been the fastest, and the tobacco epidemic could be in the most advanced stage in mainland China, forewarning what may soon happen to the country as a whole. We examined the association of smoking, with a focus on those born since 1950, with mortality in older residents in Guangzhou.

## Materials and methods

### Study subjects

All participants of the Guangzhou Biobank Cohort Study (GBCS) were recruited from 2003 to 2008. Details of the GBCS have been reported previously [12, 13]. Briefly, the GBCS is a 3-way collaboration among Guangzhou 12^th^ Hospital and the Universities of Hong Kong and Birmingham, UK. Recruitment of participants was from “The Guangzhou Health and Happiness Association for the Respectable Elders” (GHHARE), a community social and welfare organization. GHHARE is unofficially aligned with the municipal government. Membership is open to Guangzhou permanent residents aged 50 years (born after 1910) or above for a nominal fee of 4 CNY (≈50 US cents) per month. GHHARE included about 7% of Guangzhou residents in this age group, with branches in all 10 districts of Guangzhou, the capital city of Guangdong province in southern China. The baseline examination included a face-to-face computer assisted interview by trained nurses on lifestyle, family and personal medical history and assessment of anthropometrics, blood pressure, fasting plasma glucose, lipids and inflammatory markers. The Guangzhou Medical Ethics Committee of the Chinese Medical Association approved the study and all participants gave written, informed consent before participation.

### Exposure variable and potential confounders

Exposure variable was smoking, defined as having smoked at least one cigarette per day or 7 cigarettes per week for at least half a year. “Current smoker” was defined by answering “yes” to the question: “Do you smoke cigarettes now?” “Former smoker” was defined as “used to smoke but not smoking currently”. The reliability of the questionnaire was tested in 200 subjects with Kappa values of 0.88 and 0.96 for the two questions about smoking status, respectively [14].

Potential confounders included age (5-year age group), education (primary school or below, secondary school, and college or above), occupation (manual, non-manual and others), family income (<10,000, 10,000–29,999, 30,000–49,999, ≥50,000, and don’t know), physical activity assessed by the Chinese version of International Physical Activity Questionnaire (inactive, moderately active and physically active), alcohol drinking (never, former and current drinkers), self-rated health (very good, good, poor and very poor) and sex. Self-rated health was determined by the question: “In general do you think your health is very good, good, poor or very poor?” Since very few (1.6%) participants selected the extreme categories, “very good” or “very poor”, this variable was dichotomized into good (including “very good” and “good”) and poor (including “very poor” and “poor”). Occupation was assessed from participants’ longest-held occupation, categorized as manual (including the original “agricultural work”, “factory work”, or “sales and services”), non-manual (originally “administrative/managerial”, “professional/technical”, or “military/police”) and others. The correlation among the socioeconomic variables were weak (correlation coefficients ranged from 0.05 to 0.18), thus multicolinearity should not be a concern in our model.

#### Mortality

Information on underlying causes of deaths up to January 2016 was mostly obtained via record linkage with the Guangzhou Center for Disease Control and Prevention (GCDC). When participants were confirmed dead but the exact dates of death were not available, the mid-point between the date of recruitment and 31 January 2016 was used (n = 6).

### Statistical analysis

Chi-square tests or analysis of variance were used to compare participants’ baseline characteristics by smoking status. The Cox proportional assumption was checked using Schoenfeld residuals by “stphtest” command in STATA. As no evidence of violation for the proportional hazard assumption was found, the Cox proportional hazards model was used to calculate adjusted hazard ratios (HRs) with 95% CI. Group specific CI for the non-smokers HR of 1.00 was calculated to reflect the variance of the log risk in non-smokers using Plummer’s Method [15]. Participants who died of any other causes were regarded as censored at the date of death [16, 17]. For those who were alive, as registered by the police, they were right-censored on 31 January 2016. We stratified the cohort into those born in 1920–1939, 1940s and 1950s (range: 1950–1957) for analysis of all-cause mortality. We firstly conducted the analysis for each birth cohort, and then for all participants including all three birth cohorts, in men and women separately and combined.

The population attributable risk (PAR) was calculated using the following equation (in which PAR denotes the population attributable risk, P the proportion of smokers, and RR relative risk): PAR = P*(RR−1)/[P*(RR−1)+1]. The relative risk or PAR of death associated with smoking was calculated based on HRs in men and women separately. When the event rate is low, the PAR calculated by HR agrees with the approximation obtained using the standard PAR formula (i.e., using RR), which is consistent with the rare disease assumption commonly used to justify that the risk ratio and hazard ratio are equivalent when a disease is rare [18]. Information on the prevalence of smoking was obtained according to the Global Adult Tobacco Survey China 2010 Country Report (http://www.who.int/tobacco/surveillance/survey/gats/zh_gats_china_report.pdf, accessed on online on July 4^th^, 2016). The numbers of deaths attributable to smoking in men and women were then calculated by multiplying the PARs according to the relative mortality risk from the study and 2010 Population Census from the National Bureau of Statistics of China (http://www.stats.gov.cn/english/statisticaldata/censusdata/, accessed online on July 4^th^, 2016). All analysis was performed in STATA/IC 13.1.

## Results

Of the 30,430 participants at baseline, 391 were excluded because of loss to follow-up with unknown vital status and 97 excluded because of incomplete information on smoking, giving 29,942 participants (21,658 women and 8,284 men) in this paper. The mean age was 62 years (standard deviation (SD) = 7.1) years. During the average follow-up of 8.8 years (SD = 1.8), 2,986 (women 1,586 (7.3%) and men 1,400 (16.9%)) deaths were recorded.

In men, 60.3% were ever smokers. Relative to never smokers, ever smokers were younger, had lower socioeconomic position (lower education and income, and manual occupation), and tended to be physically inactive, but more commonly consumed alcohol. In women, only 3.5% were ever smokers, and, relative to never smokers they were older, had lower socioeconomic position and tended to be physically inactive (Table 1).

**Table 1:** Baseline characteristics of 21,658 women and 8,284 men aged 50+ in the Guangzhou Biobank Cohort Study first examined in 2003 to 2008 and followed up until January 2016.

	Men		Women	
	Never smokers	Ever smokers	Never smokers	Ever smokers
Number of participants (row %)	3,288 (39.7)	4,996 (60.3)	20,894 (96.5)	764 (3.5)
Age, years, mean (standard deviation)	64.7 (6.6)	64.2 (6.8)	60.9 (7)	67.2 (6.3)
Age group, years, %				
50–54	7.6	9.1	23.7	4.7
55–59	17.6	20.8	27.7	9.4
60–64	25.8	24.4	19.1	18
65–69	26.1	23.6	17.3	32.7
70–74	17.6	17.2	9.5	27.8
75–79	4.1	4	2.2	5.2
80+	1.2	0.9	0.5	2.1
Education, %				
Primary or below	22.3	35.1	46.7	79.3
Secondary	53.6	52.6	47.3	19.9
College or above	24.2	12.3	6	0.8
Occupation, %				
Manual	41.6	51.7	65.7	79.3
Non-manual	43.5	34	18.7	11.7
Others	14.9	14.3	15.6	9
Family income, CNY/year, %				
<10,000	3.6	5.5	5.9	12.3
10,000–29,999	32.2	34.9	31.3	39.5
30,000–49,999	23.1	21.4	21.3	10.2
≥50,000	21.9	16.1	16.8	6.6
Don’t know	19.2	22	24.7	31.5
Physical activity, %				
Inactive	7.5	9	8	5.9
Minimally active	43.3	45.8	39.3	43.4
Active	49.2	45.2	52.6	50.7
Alcohol use, %				
Never	65.1	46.8	79.6	70.7
Former	3.8	7.8	2.4	5
Current	31.2	45.5	18	24.4
Poor health status, %				
No	87.1	83.4	81.2	79.9
Yes	12.9	16.6	18.8	20.1

**Fig 1:**
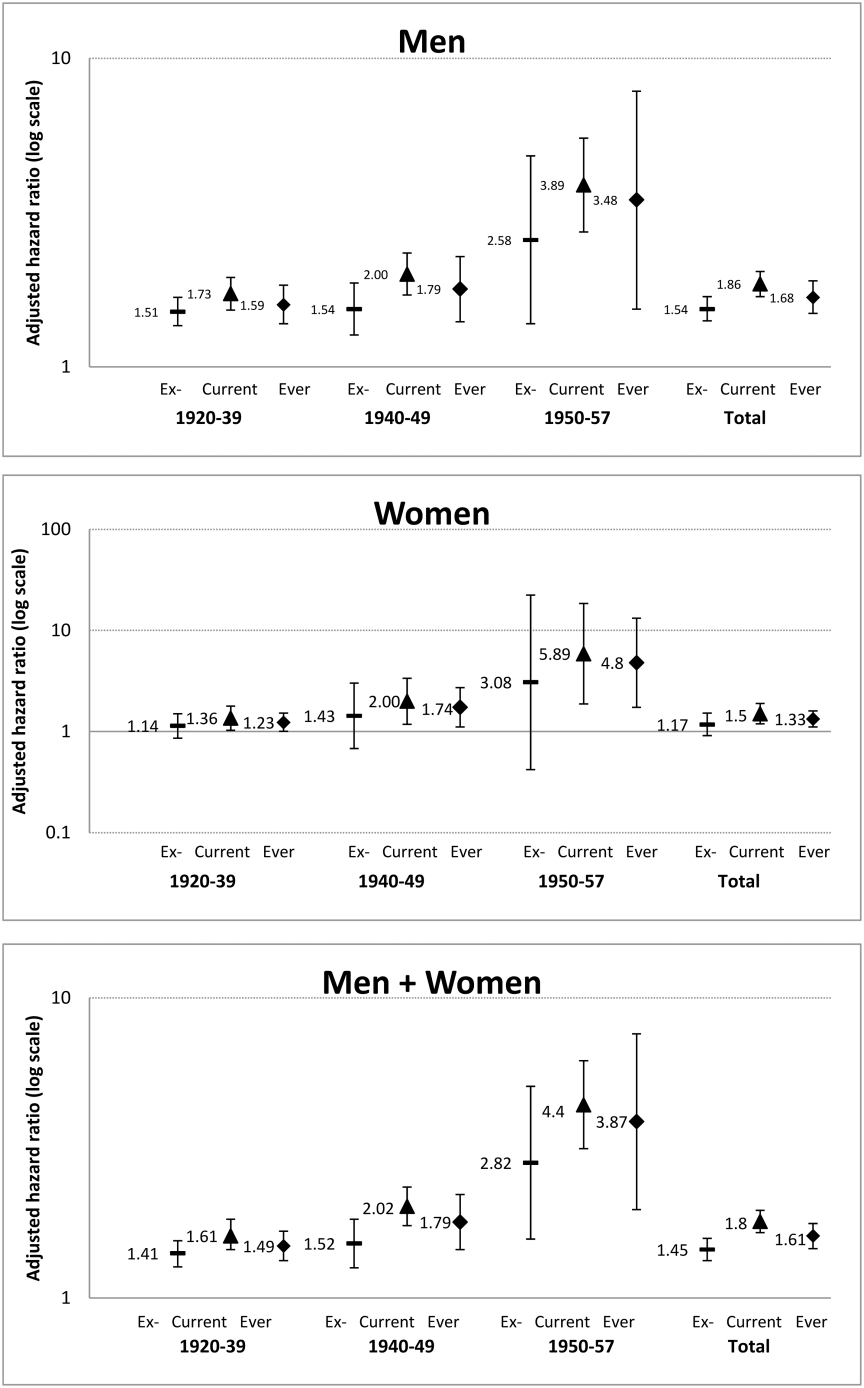
Adjusted HR and 95% CI of all-cause mortality for former (ex-), current, and ever (former+ current) smokers versus never smokers by birth cohorts in men, women and total.

Table 2 shows that for men and women who born during the 1920–1939, 1940–1949 and 1950–1957, the median (interquartile range) age at death were 77 (6), 67 (5) and 58 (4) years, respectively (Table 2). Fig 1 shows that, in men, adjusting for age, education, occupation, family income, physical activity, alcohol drinking and self-rated health, the HR of all-cause mortality in ever (current plus former) male smokers versus never smokers increased from 1.59 (1.38–1.84) in those who were born during the 1920–1939, to 1.79 (1.40–2.28) and 3.48 (1.54–7.83), in those born in the 1940s and 1950s, respectively (p for trend 0.02). A similar trend was observed in women, from 1.23 (1.004–1.52) to 1.74 (1.11–2.72), then to 4.80 (1.74–13.28), respectively (p for trend 0.12). Although women tended to show higher HR, we found no evidence that the risks varied by sex (p for sex interaction from 0.08 to 0.88). Pooling men and women together and further adjusting for sex, the HR was 4.40 (3.14–6.17) in current smokers born since 1950 (P<0.001). Former smokers showed consistently lower HR than current smokers in men and in women. Further sub-group analyses were not performed due to small numbers.

**Table 2:** Median age at death and person-years (number of deaths) by smoking status and year of birth, in 21,658 women and 8,284 men from the Guangzhou Biobank Cohort Study in 2003–2008 and followed up until January 2016.

Year of birth	Age at death (median, IQR), years	Person-years (No. of deaths) by smoking status		
		Never	Former	Current
Men				
1920–1939	77, 6	15343 (309)	11430 (367)	8344 (286)
1940–1949	68, 5	13455 (98)	9218 (111)	11926 (170)
1950–1957	58, 5	3479 (8)	1783 (11)	4074 (37)
Women				
1920–1939	77, 6	59173 (924)	2537 (54)	2437 (57)
1940–1949	67, 5	89850 (415)	829 (8)	1285 (16)
1950–1957	58, 3	57511 (103)	158 (1)	267 (3)
Total				
1920–1939	77, 6	74516 (1233)	13968 (421)	10782 (343)
1940–1949	67, 5	103306 (513)	10047 (119)	13211 (186)
1950–1957	58, 4	60991 (111)	1942 (12)	4341 (40)

## Discussion

In this cohort study of older Chinese who underwent the most advanced stage of tobacco epidemic in mainland China, we observed in all participants increasing mortality risk in ever smokers versus never smokers from 1.49 in those born in1920-1939, to 3.87 in those born since1950. Because of the risk reduction from quitting, HR for ever smoking would under-estimate the risk associated with smoking. In current smokers, the HR of 4.40 (95% CI 3.14–6.17) was very high, but the lower limit of the 95% CI of 3.14 supports that the HR in men has reached a level well over three. Such a large relative risk observed in the lower limit of the 95% CI has never been reported in Chinese cohorts previously. The CKB also showed that the few urban men who had started smoking before the age of 15 years were at even greater risk (RR 2.64, 95% CI 2.19–3.19) [7]. Hence, our relative risk of greater than three in a cohort born more recently is quite plausible. Our results further suggest that the tobacco epidemic could have reached a more advanced stage in Guangzhou than other places of China reported in the CKB and possibly the rest of mainland China [7, 10, 19–21].

Guangzhou is close (about 120 km) to Hong Kong, which has an advanced stage of tobacco epidemic [22] and a mortality relative risk of about 2 in people aged 65 years or more [23]. But participants of the Hong Kong cohort were recruited in 1998–2001 and born in the 1930s [23, 24]. In mainland China, the relative mortality risk in the CKB (1.65) and most of the earlier cohorts were lower than 2 [10, 11, 19–21, 25]. A notable exception was a small cohort of 1,696 factory workers in Xi’an, which showed a relative risk of 2.42 (95% CI 1.72–3.42) in men and 2.32 (95% CI 1.18–4.56) in women for ever versus never smokers after a very long mean follow-up of 20 years [9]. However, the lower limits of the 95% CI were below two. Studies with longer follow up would yield greater relative risks, as shown clearly by the British Doctors Cohort Study [26, 27]. In the US, where the epidemic has come to the final stage (i.e., stage 4, the decline in smoking prevalence is evident in this stage), the smoking-related mortality risk in smokers versus non-smokers has increased to about 3 over the last 50 years [28]. The increased relative risk in smokers versus non-smokers could be due to the increased incidence of smoking-related disease and/or reduction in competing risk such as deaths from infectious disease in the past several decades. Also, the increase life expectancy of people born after the establishment of People’s Republic of China in October 1949 could have benefited non-smokers much more than persistent smokers starting at younger age, resulting in a widening the gap of the survival curves and increasing relative risk. New and large cohort studies of people born since 1950 or post World War II with longer follow-up are needed to assess the increasing risk of mortality associated with smoking in China and elsewhere. The increasing risk and growing epidemic in other populations, especially in the big cities where the tobacco epidemic is more advanced, in China and other rapidly developing countries in the next few decades needs to be closely monitored. Longer follow up of existing cohorts and new cohort studies including more recently born cohorts will help clarify the picture.

Our focus on those born after the birth of PRC has shown an alarming finding. These people had not gone through World War II and the civil war (1946–49), which should imply longer survival but could be compromised by greater cigarette consumption. The high RR of premature death in middle age from smoking in this group means that smoking would lead to premature death, disease and economic burdens very soon and in an unexpectedly large scale in many middle aged, as well as older people, posing huge challenges to the unprepared health care and related systems. To estimate absolute numbers of tobacco-attributed deaths in China in 2010, the smoking-attributed fractions of all deaths in our study have been applied to numbers of deaths in men and women separately in mainland China at ages 30 year or older. This shows that there were about 1.5 million smoking-attributed deaths in 2010 (1,360,000 males, 146,000 females; S1 Table), 50% higher than those estimated by the CKB [29]. The numbers should be higher now (i.e., in 2017). If the current pattern of smoking is sustained, and the compliance to the Framework Convention on Tobacco Control (World Health Organization’s first international treaty ratified by almost all countries to address a public health problem) [30] continues to be lagging behind, China will not be able to meet the United Nations 2030 Sustainable Development Goals (SDG) to reduce premature deaths (before age 70) from non-communicable diseases (NCDs) by one-third.

Our study has important clinical and public health implications. The World Health Organisation statement that “tobacco kills up to one in every two users” [31] is derived from Peto based on an RR of two for all-cause mortality from smoking [32]. This statement now appears to be an under-estimate, at least for countries like the US, UK and Australia, or large cities such as Guangzhou with an RR over two, and approaching or already three. Translating such relative risk means that the WHO statement could be revised to “Tobacco kills *at least* one in every two, or even two in every three users”. Absolute risk, which means the chance of developing a disease (or death) during a specified period, is easier to understand than relative risk. Most smokers and many physicians grossly under-estimate the absolute mortality risks of smoking. Physicians’ and other health professionals’ warning to their smoking patients, healthy clients or the public using an absolute risk of at least one out of two, or even as high as two out of three for high risk smokers could be more striking and effective in motivating more smokers to quit.

Our results on the benefits in former smokers regardless of the year of birth and sex are consistent with previous studies in China and elsewhere. Hence, quitting for all smokers in both sexes respective of age must be the most important action to reduce the death toll. Pooling former and current smokers into ever smokers will underestimate RR. However, excluding former smokers would under-estimate the absolute attributable numbers or burden of disease, as some former smokers will still die from smoking-related conditions and they should thus still be counted. The benefits of quitting smoking as observed in former smokers are likely diluted by reverse causality, i.e., some quit directly because of smoking-related illness. Future studies should enquire about reasons of quitting, due to choice or illness, and examine changes in smoking behaviour during follow up and subsequent health outcomes.

Our study has other limitations. First, the sample size was relatively small, which limited detailed subgroup analysis. Second, our sample may not be fully representative of the entire older population in China and as other population based cohort studies of older people, women were oversampled. However, within our sample, the participants had fairly similar levels of chronic diseases such as diabetes and hypertension to nationally representative samples of urban Chinese [12]. Finally, our study of people aged 50 years or older could have underestimated the risk of smoking in the younger populations who start smoking as teenagers and may thus have higher lifetime cumulative exposure, unless they stop early.

In conclusion, the mortality relative risk could have reached three in smokers born after 1949 in Guangzhou and other areas which have the longest history of smoking, as in the UK, US and Australia. If confirmed, this is a more striking warning that China will be facing an even larger public health issue of tobacco deaths than hitherto forecast, unless China quickly and strictly complies with the Framework Convention on Tobacco Control.

## Supporting information

S1 Table Number of deaths attributed to smoking in China in 2010. (DOCX)
