# Supplementary material for: High relative risk of all-cause mortality attributed to smoking in China: Guangzhou Biobank Cohort Study
Source: PLoS One. 2018 Apr 26;13(4):e0196610. doi: 10.1371/journal.pone.0196610 (PMC5919701; doi:10.1371/journal.pone.0196610)
Supplement: S1 Table — (DOCX) [file pone.0196610.s001.docx]

S1 Table. Number of deaths attributed to smoking in China in 2010

|  | Male |  | Female |  | Both |  |
| --- | --- | --- | --- | --- | --- | --- |
|  | Number of deaths^*^ | Number of deaths attributed to smoking^†^ | Number of deaths^*^ | Number of deaths attributed to smoking^†^ | Number of deaths^*^ | Number of deaths attributed to smoking^†^ |
| 30-64 | 1,411,517 | 705,759 | 705,754 | 27,144 | 2,117,271 | 732,903 |
| 65+ | 2,683,934 | 650,651 | 2,313,024 | 119,337 | 4,996,958 | 769,987 |
| Total (30y+) | 4,095,451 | 1,356,409 | 3,018,778 | 146,481 | 7,114,229 | 1,502,890 |

Data are number of deaths.

*: 2010 Population Census from the National Bureau of Statistics of China (http://www.stats.gov.cn/english/statisticaldata/censusdata/, accessed online on July 4th, 2016).

^†^: Estimated by applying relative mortality risk of 3.0 for deaths at age 30–64 years, and 1.8 for deaths at aged 65+ years.
